# Antitoxin

**Published:** 1897-12

**Authors:** 


### Antitoxin.

Henry R. Slack, Ph. D., M. D., of LaGrange, Georgia, expresses (*Atlanta Medical and Surgical Journal*, November, 1897,) a high opinion of antitoxin as a preventive and curative agent in the treatment of diphtheria. His own experience in private practice bears out fully the favorable reports made to the American Pediatric Society by its special committee, and the clinical cases recited by him testify to the prompt and powerful action exerted by antitoxin on both the local and constitutional symptoms of the disease. Dr. Slack concludes that the anti-diphtheritic serum should be administered freely and promptly, as the loss of twenty-four hours may mean the sacrifice of a life. He recommends doses of 1000 to 2000 units, according to the severity of the case, to be repeated in twenty-four hours if improvement is not manifest. A third dose may be given with perfect safety. Dr. Slack urges the use of the most concentrated strength of an absolutely reliable preparation. In his own cases he used diphtheria antitoxin of both foreign and domestic manufacture. He prefers that prepared by Parke, Davis & Co., for two reasons: 1. It is put up in hermetically sealed, large-necked, glass bulbs. 2. It can be obtained fresher, thus increasing its reliability.

Dr. Slack's article bears out the many tributes that have been paid to the anti-diphtheritic serum of Parke, Davis & Co. on the score of its entire freedom from fatalities, casualties or complications of any kind; its great concentration, and the promptness with which its curative powers are manifested.

---

Dr. Byron Robinson, of Chicago, gives the following direction for a vaginal douche:

1. Use a fountain syringe holding three gallons of water, with a four foot head.
2. Begin with (married women) three quarts of boiled water at 103°.
3. Increase the heat one degree at each sitting until it is as hot as can be borne.
4. Increase the amount of the douche one pint each time, until three gallons are taken.
5. Use the douche in the morning, and in the evening when retiring.

6. The duration of a three-gallon douche should be twenty-five minutes.

7. The patient should lie on the back, with the thighs flexed on the abdomen, and the legs flexed on the thighs.

8. The douche should be taken on a level plane, the ironing board serving a good purpose, and not in the bed, on the water closet or in the bath tub.

9. The douche should never be taken in a standing or sitting posture.

10. A handful of salt (Na. Cl.) and a teaspoonful of alum may be added to every gallon, the salt to prevent reaction and the alum to astringe and check waste by secretion.

11. The vaginal tube used in giving the douche should be sterilized, and every patient should have her own tube.

12. A vaginal douche given according to the above directions will prove to be of much therapeutic value in the treatment of pelvic diseases, an agent to prevent disease, and a great comfort to the patient.

---

**Dr. F. B. McRhea,** has removed from Temple to 212 Binz building, Houston, Texas.

---

**Dr. R. B. Henderson,** has removed from Richardson to Bagwell, Texas.

---

**The Committee** appointed by theconference of the State Board of Health to memoralize Congress to appoint a commission to study yellow fever in Havana, have drawn up the following, and will submit it to Congress:

"Be it enacted by the Senate and House of Representatives of the United States of America in Congress assembled:

Section 1. A commission of experts shall be appointed by the president for the purpose of making investigations relating to the cause and prevention of yellow fever.

Section 2. This commission shall consist of four expert bacteriologists, one to be detailed from among the medical officers of the army, and one from among the medical officers of the navy, one from among the medical officers of the Marine Hospital Service, and one to be appointed from civil life.

Section 3. The president shall name one of the members of the commission as chairman, who shall direct the work of the commission and report to him from time to time the results attained. The headquarters of the commission shall be in Washington, D. C., and one or more of the members shall be detailed to conduct investigations in the city of Havana, Cuba, or in some other locality where yellow fever prevails.

SECTION 4. The medical officers of the army, the navy and the Marine Hospital Service detailed as members of this commission shall receive no compensation beyond their salaries. But during the time that they are necessarily absent from their proper stations in the performance of the duties imposed upon them by this act, their necessary living and traveling expenses shall be paid from the appropriation made in this act. The civilian member of the commission shall receive, in addition to his necessary living and traveling expenses, six dollars per day during the time he is actually employed in prosecuting the investigation comtemplated in this act.

SECTION 5. The sum of twenty thousand dollars is hereby appropriated out of any money in the treasury of the United States not otherwise appropriated, for carrying out the provisions of this act and for the purchase of necessary instruments and material, for rent of a laboratory in Havana, Cuba, or elsewhere, and other incidental expenses.

We don't like it: the army and navy representatives should receive extra pay for this service and the entire commission should be paid for the whole time they are away from home $10 a day each and expenses, whether "actively employed" or not. As it is, it is not a very tempting office.

